# Bidirectional interactions along the microbiota–gut–brain axis in radiation-induced brain injury: mechanisms and therapeutic insights

**DOI:** 10.3389/fonc.2026.1774021

**Published:** 2026-02-05

**Authors:** Yayi Yuan, Nan Wang, Kang Ning, Xuhong Dang

**Affiliations:** 1 Key Laboratory of Molecular Biophysics of the Ministry of Education, Hubei Key Laboratory of Bioinformatics and Molecular-imaging, Center of AI Biology, Department of Bioinformatics and Systems Biology, College of Life Science and Technology, Huazhong University of Science and Technology, Hubei, China; 2 China National Nuclear Corporation (CNNC) Key Laboratory for Radiation Protection Technology, Shanxi Key Laboratory for Radiation Safety and Protection, Department of Radiology and Environmental Medicine, China Institute for Radiation Protection, Taiyuan, China

**Keywords:** brain tumors, gut microbiota, microbiota–gut–brain axis, radiation therapy, radiation-induced brain injury

## Abstract

Radiation therapy is widely used for the treatment of brain tumors and metastases from extracranial malignancies; however, it may also cause damage to normal brain tissue, potentially resulting in radiation-induced brain injury (RBI). Emerging evidence highlights the microbiota–gut–brain axis (MGBA) as a critical mediator of bidirectional communication between the gut microbiota and the brain, playing an important role in central nervous system (CNS) homeostasis and pathology. This review aims to summarize current evidence regarding the potential involvement of the MGBA in the pathogenic mechanisms of RBI, with particular emphasis on bidirectional interactions along this axis. We focus on underlying mechanisms, including neuroimmune and inflammatory responses, signal transduction, DNA damage, and oxidative stress. By integrating these perspectives, this review seeks to provide a novel conceptual framework for understanding RBI and to identify potential directions for future MGBA-targeted interventions.

## 1 Introduction

Radiation therapy is a cornerstone in the treatment of malignant tumors and is widely applied in the management of primary brain tumors and metastases from extracranial malignancies (1). In the United States alone, more than 200,000 patients undergo whole-brain irradiation each year (2). Although cranial irradiation is effective for tumor control, it inevitably exposes adjacent normal brain tissue to radiation, which may result in radiation-induced brain injury (RBI). RBI is primarily characterized by cerebral edema, demyelination, cognitive impairment, memory deficits, and other functional disturbances (3, 4). Notably, 30%–50% of survivors following whole-brain radiotherapy develop clinically significant cognitive decline, which profoundly compromises central nervous system (CNS) function and severely affects long-term quality of life (5–8).

The pathogenesis of these effects involves a dynamic interplay of multiple biological processes. The prevailing view holds that RBI is driven by interactions among vascular injury, neuroinflammation, cellular dysfunction, and molecular disturbances (4, 7, 9, 10). A central mechanism involves the concurrent effects of radiation on brain microvascular endothelial cells and microglia, leading to disruption of the blood–brain barrier (BBB) and early-stage neuroinflammation, which together form a vicious cycle. The resulting chronic inflammatory milieu promotes neuronal apoptosis and synaptic dysfunction and triggers toxic responses in astrocytes and oligodendrocytes, ultimately leading to demyelination. Activation of signaling pathways associated with oxidative stress and nuclear factor kappa B (NF-κB), together with the engagement of emerging cell death pathways such as ferroptosis, collectively forms a complex injury network. This network ultimately results in cognitive impairment and other long-term or irreversible neurological deficits.

This complex injury network constitutes the pathological basis of RBI. In recent years, in addition to the mechanisms described above, increasing attention has been directed toward the role of the microbiota–gut–brain axis (MGBA). The gastrointestinal tract harbors a vast community of commensal microorganisms, currently estimated to be approximately equal in number to human cells in the body (11). A bidirectional regulatory relationship exists between this microbial community and the brain. On the one hand, the brain exerts “top-down” regulation over gastrointestinal motility, sensation, secretory function, and local immune status via the vagus nerve and neuroendocrine pathways (12–15). On the other hand, the gut microbiota influences brain function, emotion, cognition, and BBB integrity in a “bottom-up” manner through neural, immune, endocrine, and metabolic pathways, mediated by microbial metabolites, neuroactive substances, and immunomodulatory signals (16–18).

Importantly, ionizing radiation, while inducing brain injury, can also indirectly disrupt the intestinal microenvironment, leading to gut microbial dysbiosis (19). The disturbed gut microbiota may subsequently act as a persistent source of pathological signals, exacerbating central neuroinflammation, impairing neural repair, and aggravating cognitive dysfunction through ascending MGBA pathways (16, 20). Research by Cui et al. has provided compelling evidence that the gut microbiota plays a critical role in the initiation and progression of radiation-induced injury. Their study demonstrated that transplantation of fecal microbiota from healthy donor mice into irradiated mice significantly improved survival and promoted structural and functional repair of the damaged intestinal epithelium. In contrast, transplantation of feces from mice with radiation proctitis into germ-free mice markedly increased susceptibility to intestinal radiation injury (21).

Nevertheless, the specific mechanisms underlying bidirectional MGBA interactions in the context of RBI remain incompletely understood. This review aims to explore the potential mechanisms linking the gut microbiota and RBI via the MGBA, focusing on immunity and inflammation, signal transduction, DNA damage, and oxidative stress. A clearer understanding of these mechanisms may provide a foundation for the development of novel therapeutic strategies for RBI.

## 2 Immunity and inflammation

The immune system maintains a symbiotic relationship with the gut microbiota to preserve host homeostasis (22, 23). These microbial communities participate in host metabolic processes, promote the maturation of intestinal immune cells, and contribute to the maintenance of internal physiological balance (24, 25).

### 2.1 Impact of RBI on the intestinal tract and microbiota

RBI may exert a significant “top-down” influence on distal intestinal homeostasis through the MGBA, primarily manifested as direct damage to intestinal structure and barrier function, as well as disruption of the intestinal microenvironment and microbial community stability.

First, RBI induces intestinal structural injury and barrier dysfunction (26–29). Experimental studies have shown that mice exposed to a single dose of 15 Gy whole-brain irradiation develop pathological alterations, including damaged intestinal epithelial cells, reduced villus number and length, and disrupted intercellular junctions, resulting in severe impairment of intestinal barrier integrity (26, 28). This breach of the physical barrier predisposes the intestine to subsequent microbial dysbiosis and immune activation. Notably, supplementation with Lactobacillus reuteri significantly alleviated pathological intestinal damage in irradiated mice and reduced *in vivo* levels of key pro-inflammatory cytokines, including tumor necrosis factor-α (TNF-α) and interleukin-6 (IL-6), providing further evidence for the critical involvement of intestinal injury and inflammatory responses in RBI (30, 31).

Second, RBI may induce alterations in the intestinal microenvironment through neuroendocrine and inflammatory mediators. Following cranial irradiation, activated glial cells and damaged tissues release a range of inflammatory signals that act on the intestine via the circulatory system or neural pathways (20, 27, 32, 33). Specifically: (1) Pro-inflammatory cytokine–driven disruption. Studies have demonstrated that blockade of TNF-α signaling with anti–TNF-α antibodies significantly alters gut microbiota composition and markedly attenuates intestinal inflammation, indicating that TNF-α exacerbates intestinal inflammatory responses and modulates microbial composition (34). (2) Impaired anti-inflammatory and repair functions. Under physiological conditions, the gut microbiota induces a mild inflammatory response that stimulates regulatory T cells to produce interleukin-10 (IL-10) (35). IL-10 subsequently exerts anti-inflammatory effects, protecting the host from excessive inflammation while supporting the survival of the gut microbiota (13, 35). IL-10 deficiency leads to severe microbial dysbiosis; studies have shown that IL-10–deficient mice exhibit expanded populations of Firmicutes and Bacteroidetes, with Escherichia coli increasing by approximately two orders of magnitude, suggesting that the role of IL-10 in maintaining microbial homeostasis after RBI may be compromised (36). (3) Dysregulation of key immune modulation. Transforming growth factor–β (TGF-β) plays a critical role in maintaining gut microbiota stability. Loss of TGF-β signaling results in an abnormal increase in Enterobacteriaceae, particularly E. coli, within dendritic cells of the intestinal lamina propria (27, 33, 37, 38).

**Figure 1:**
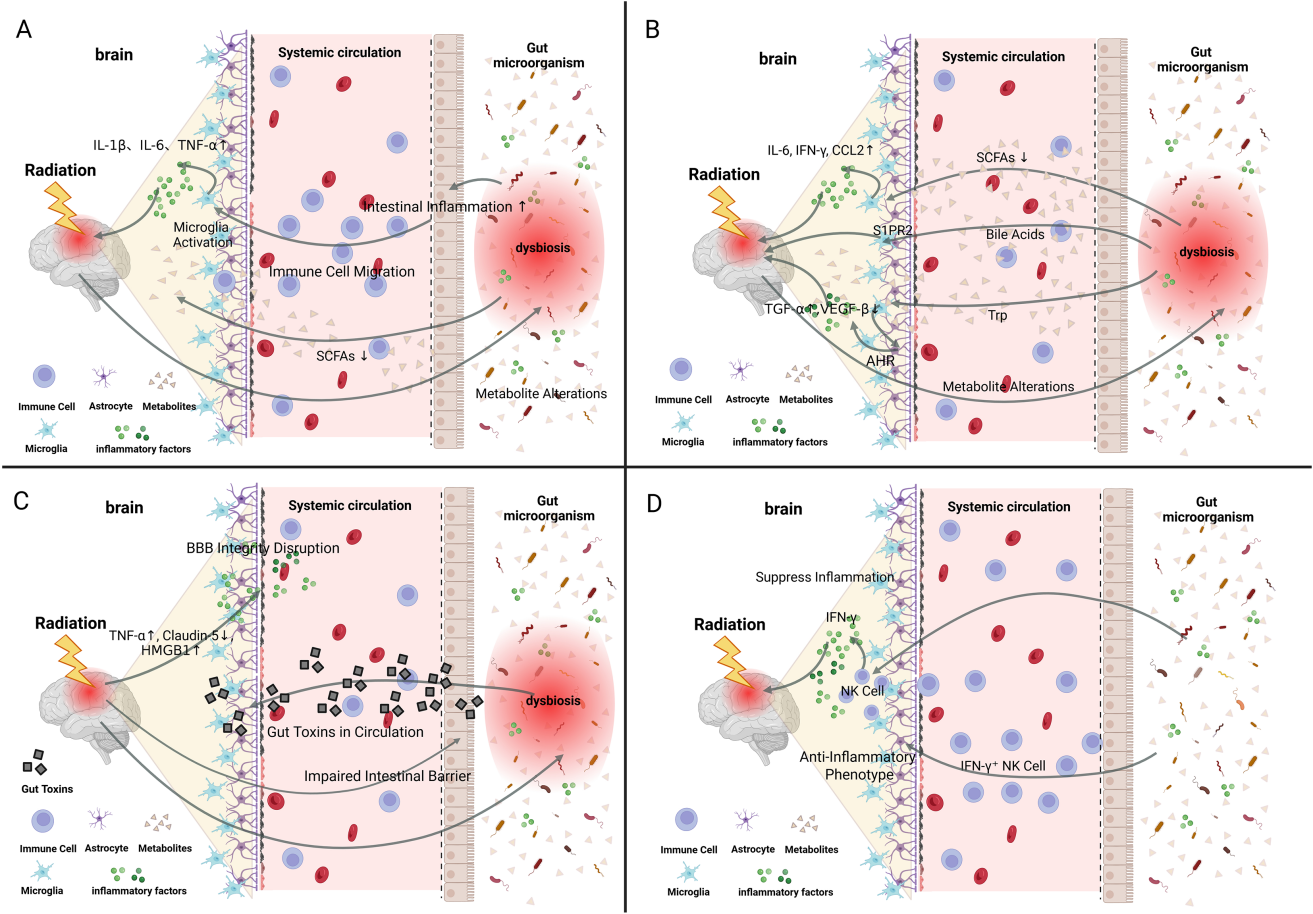
Role of the gut–brain axis in immune inflammation in radiation brain injury. **(A)** Gut microbiota and metabolites (e.g., SCFAs) maintain microglial homeostasis. Post-RBI gut dysbiosis and BBB disruption promote peripheral immune cell infiltration and inflammatory mediator access, triggering aberrant M1 polarization of microglia. This leads to neurotoxic cytokine release (IL-1β, IL-6, TNF-α), suppressed neurogenesis, and cognitive dysfunction. **(B)** SCFAs (butyrate/propionate) regulate microglial maturation/function via HDAC1 inhibition and exert anti-inflammatory effects; RBI-induced SCFA decline exacerbates neuroinflammation. Primary bile acids activate S1PR2 to promote neuroinflammation. Tryptophan derivatives activate microglial AHR, promoting astrocytic TGF-α and inhibiting VEGF-β to alleviate CNS inflammation. **(C)** Ionizing radiation directly damages BBB (endothelial injury, claudin-5 downregulation, HMGB1 release), increasing permeability. RBI-associated gut dysbiosis elevates circulating gut-derived factors (e.g., LPS). These factors cross the impaired BBB into the CNS, forming a toxic inflammatory milieu and exacerbating the neuroinflammation vicious cycle. **(D)** Gut microbiota promotes the maturation of meningeal NK cells to produce anti-inflammatory IFN-γ. These gut-licensed IFN-γ^+^ NK cells migrate into the brain parenchyma, inducing astrocytes to polarize into the anti-inflammatory LAMP1^+^TRAIL^+^ phenotype and thereby suppressing CNS inflammation. Created in BioRender. Jiejie, W. (2026) https://BioRender.com/necagsv.

In summary, RBI initiates a “descending” pathway along the MGBA. Injury to neurons or glial cells alters the cerebral microenvironment, leading to the production of inflammatory mediators that indirectly disrupt intestinal function and gut microbiota homeostasis (20, 33) (**Figure 1A**). Inflammatory factors serve as key molecular mediators in this process.

### 2.2 Influence of the gut microbiota on RBI

The gut is the largest immune organ in the human body, harboring approximately 70%–80% of total immune cells. The gut microbiota and its metabolites can influence the CNS through multiple “bottom-up” pathways, including direct interactions with intestinal immune cells or neural fibers, as well as indirect modulation of brain function through the secretion of microbial metabolites (20, 33, 39, 40). Intestinal dysbiosis induced by RBI can, in turn, exacerbate neuroinflammation and brain injury through several mechanisms.

#### 2.2.1 Aberrant activation of microglia

Microglia, the primary innate immune cells of the CNS, have their maturation, morphology, and functional homeostasis regulated by the gut microbiota and its metabolites (41). Among these, microbial-derived short-chain fatty acids (SCFAs), which reach the brain via the circulatory system, are essential for normal microglial development and functional maintenance (41, 42). Studies have shown that microglia in germ-free or antibiotic-treated mice exhibit developmental immaturity and functional impairment, underscoring the critical role of the gut microbiota in shaping central immune cell function (20, 33, 43, 44)(**Figure 1A**).

A stable gut microbial community is fundamental to maintaining physiological microglial function, and its disruption can exacerbate neural injury (45). However, gut dysbiosis following RBI perturbs this homeostasis. Microbial imbalance results in reduced levels of beneficial metabolites, such as SCFAs, and conveys persistent injury signals to the brain through enhanced intestinal and systemic low-grade inflammation. For example, Citrobacter-induced intestinal infection has been shown to aggravate microglia-mediated neuroinflammation, leading to expansion of brain lesions (45). In addition, intestinal dysbiosis intensifies gut inflammation, facilitating the migration of peripheral immune cells into the brain, where they interact with resident microglia and other neural cells to collectively amplify the cerebral immune response (20, 46)(**Figure 1A**).

Following RBI, disruption of the BBB permits peripheral inflammatory mediators to infiltrate the brain and activate microglia (4, 6, 33, 47, 48). Aberrantly activated microglia predominantly polarize toward the pro-inflammatory M1 phenotype and release large quantities of neurotoxic cytokines, including IL-1β, IL-6, and TNF-α (4, 49, 50). These cytokines inhibit the proliferation and differentiation of neuronal precursor cells and impair neurorepair processes. Such polarization represents not only a marker of inflammation but also an effector mechanism that drives neuronal injury, suppresses hippocampal neurogenesis, and ultimately leads to cognitive dysfunction (51) (**Figure 1A**).

Animal studies have demonstrated that rats exposed to a cumulative dose of 40 Gy (administered as 5 Gy fractions twice weekly via whole-brain ¹³^7^Cs γ-irradiation for four consecutive weeks) exhibit persistent microglial activation and sustained TNF-α release, accompanied by hippocampal dysfunction, for up to six months (52). These findings indicate that aberrant microglial activation can persist long after radiation exposure and is closely associated with long-term cognitive impairment, providing an important basis for chronic neurological deficits (**Figure 1A**).

Depletion or inhibition of hyperactivated microglia has been shown to alleviate post-radiation neuroinflammation and cognitive impairment (32). In addition, remodeling of the central immune milieu through transplantation of brain macrophages with protective properties can exert neuroprotective effects. In C57BL/6J mice subjected to whole-brain irradiation (three fractions of 3.3 Gy of ¹³^7^Cs γ-rays delivered at a dose rate of 2.58 Gy/min), replacement of resident microglia with engrafted brain macrophages protected against long-term cognitive deficits induced by whole-brain radiotherapy (40). Furthermore, certain probiotics, through modulation of the gut microbiota, inhibition of microglial activation, and reduction of pro-inflammatory cytokine levels, represent a potential upstream intervention strategy for RBI via the MGBA (53).

#### 2.2.2 Changes in microbial metabolites

The gut microbiota produces a variety of neuroactive metabolites that function as critical signaling molecules mediating ascending influences along the MGBA (**Figure 1B**).

SCFAs: Butyrate, propionate, and related SCFAs play indispensable roles in regulating microglial maturation and function (54). Studies using germ-free mouse models have confirmed that SCFA deficiency leads to immature microglial development, aberrant morphology, and functional impairment, whereas exogenous SCFA supplementation can reverse these abnormalities (48, 55). Propionate and butyrate may inhibit histone deacetylase 1 (HDAC1) activity, thereby increasing histone H3 acetylation, which contributes to the normalization of microglial morphology and reduction in the expression of inflammatory mediators such as IL-6, IFN-γ, and CCL2 (56). SCFAs also exert direct anti-inflammatory effects. For example, butyrate downregulates the expression of pro-inflammatory genes within the intestinal immune system, and a reduction in butyrate-producing bacteria is closely associated with a pro-inflammatory state (57, 58). It is therefore hypothesized that a decline in SCFA levels following RBI may weaken endogenous anti-inflammatory capacity, thereby exacerbating neuroinflammation (**Figure 1B**).

Bile Acids: The gut microbiota can convert primary bile acids into secondary bile acids, which constitute an important class of signaling molecules. These bile acids can modulate neuroimmune responses through the activation of specific receptors. For example, conjugated bile acids (e.g., taurocholic acid) can activate sphingosine-1-phosphate receptor 2 (S1PR2), thereby promoting neuroinflammation under pathological conditions (59). In contrast, secondary bile acids (e.g., deoxycholic acid) can activate G protein-coupled bile acid receptor 5 (TGR5), which inhibits microglial activation and consequently exerts anti-inflammatory effects (60, 61). RBI-induced dysbiosis may disrupt the bile acid metabolic profile, thereby disturbing the balance between neuroprotective and neurotoxic effects mediated by different receptors and ultimately influencing disease progression (**Figure 1B**).

Tryptophan Derivatives: Indole compounds produced through microbial metabolism act as endogenous ligands for the aryl hydrocarbon receptor (AHR). Activation of the AHR signaling pathway in microglia enables communication with neighboring astrocytes. These signals can promote the production of protective transforming growth factor-alpha (TGF-α) while simultaneously inhibiting the production of vascular endothelial growth factor-beta (VEGF-β) in astrocytes, collectively attenuating CNS inflammation (61) (**Figure 1B**).

#### 2.2.3 Potential exacerbating effects on the impaired BBB

BBB is a critical interface between the circulatory system and the CNS. The gut microbiota can influence BBB permeability by regulating the expression of tight junction proteins (62, 63). In addition, the microbiota can modulate the development and maintenance of the BBB through epigenetic mechanisms mediated by metabolites such as SCFAs (41)(**Figure 1C**).

Disruption of the BBB by ionizing radiation is a dynamic process that is potentially associated with the release of various substances following radiation exposure (64–66). Radiation-induced endothelial cell damage alters vascular permeability, thereby compromising BBB integrity and triggering inflammatory responses (57, 65). Early BBB disruption induced by radiation is closely associated with TNF-α activation and the downregulation of the tight junction protein claudin-5 (67). High-mobility group box 1 (HMGB1) is activated and passively released by macrophages and monocytes into the extracellular environment, further increasing endothelial permeability (68, 69)(**Figure 1C**).

Based on the evidence that radiation directly compromises BBB integrity (57, 63, 65–67, 70), a plausible mechanistic hypothesis is proposed. Gut dysbiosis frequently associated with RBI may elevate systemic levels of gut-derived neuroactive metabolites and microbial products (e.g., lipopolysaccharide). Concurrently, radiation-induced BBB damage and increased permeability may facilitate the enhanced entry of these circulating factors into the CNS. The translocation of such substances may contribute to a toxic inflammatory milieu within the brain, disrupting immune homeostasis, thereby exacerbating a vicious cycle of neuroinflammation (70).

#### 2.2.4 Remote regulation of central inflammation by the gut microbiota

The gut microbiota can also exert finely tuned negative regulation of central inflammation through the modulation of specific immune cell populations. For instance, the gut microbiota can regulate the maturation of natural killer (NK) cells in the meninges, promoting their production of anti-inflammatory interferon-gamma (IFN-γ) (71). These “gut-licensed” IFN-γ^+^ NK cells subsequently migrate into the brain parenchyma and induce the transformation of astrocytes into a specialized anti-inflammatory phenotype (LAMP1^+^TRAIL^+^) (71). This astrocyte subtype can markedly suppress inflammatory responses within the CNS, revealing a unique ascending pathway through which the gut microbiota influences central immune regulation(**Figure 1D**).

In summary, a bidirectional and self-amplifying pathological loop is established between RBI and the gut microbiota via the MGBA. In the descending pathway, RBI disrupts intestinal barrier integrity and immune homeostasis through brain-derived inflammatory and stress-related signals, leading to microbial dysbiosis. In the ascending pathway, the dysbiotic gut microbiota feeds back to exacerbate central neuroinflammation, neuronal injury, and cognitive dysfunction through mechanisms including aberrant microglial activation, altered neuroactive metabolite profiles, aggravated BBB damage, and remote immune regulation. This cyclical amplification mechanism underscores the central role of the MGBA in the pathogenesis of RBI and highlights potential therapeutic strategies and intervention targets for the prevention and treatment of RBI through modulation of the gut microbiota (e.g., probiotics, prebiotics, or specific metabolites).

## 3 Signal transduction disruption

**Figure 2:**
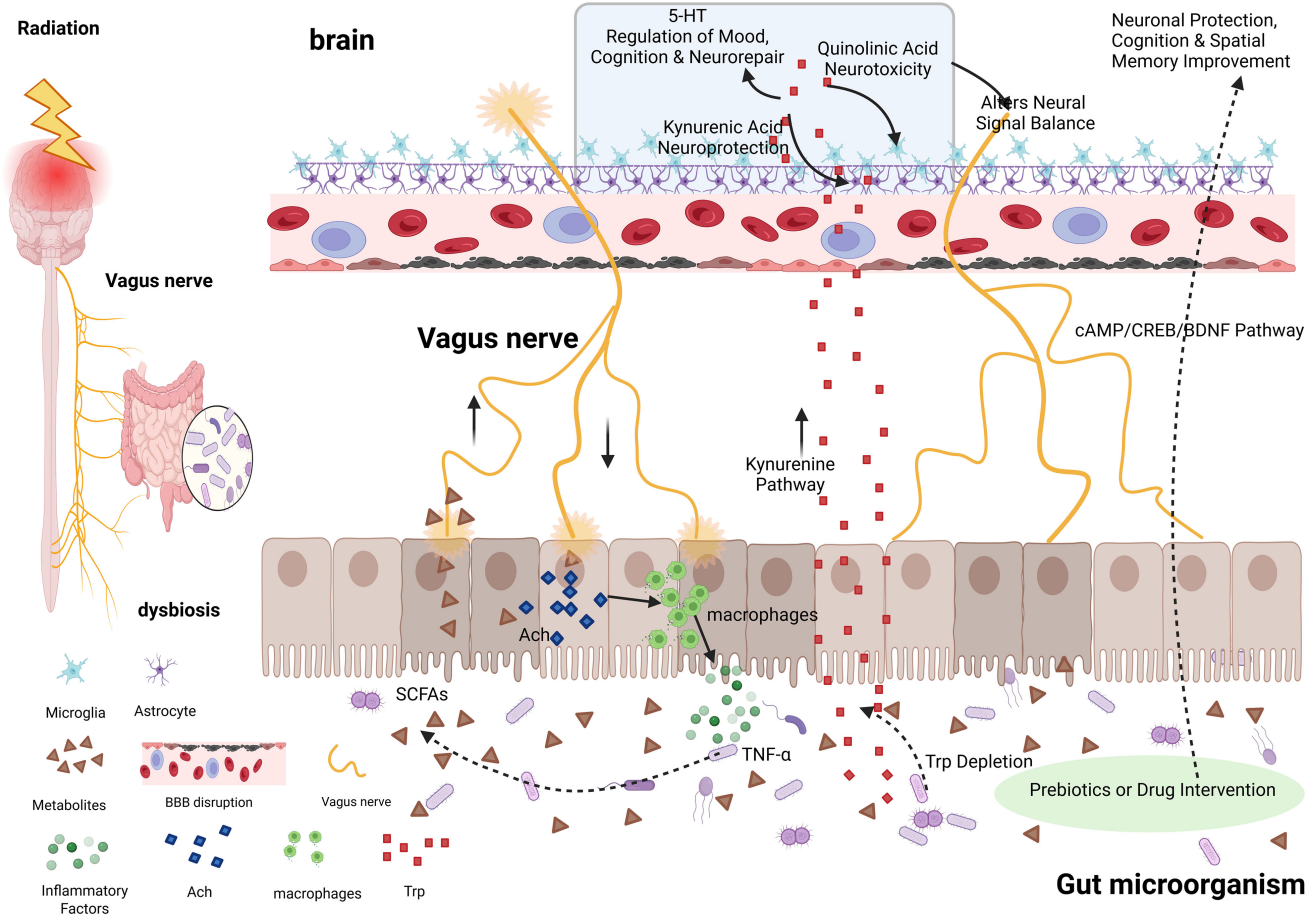
Role of the gut–brain axis in signaling pathways in radiation brain injury. (1) Vagus nerve: Afferent fibers sense microbial metabolites (e.g., SCFAs) and transmit signals to the brain; efferent fibers inhibit intestinal inflammation via cholinergic anti-inflammatory pathway, regulating gut microbiota. (2) Tryptophan metabolism: Kynurenine pathway produces neuroprotective kynurenic acid (astrocytes) and neurotoxic quinolinic acid (microglia), balanced by gut microbiota. (3) Intervention potential: Prebiotics/drugs (e.g., total glucosides of paeony) modulate gut microbiota, activate cerebral cAMP/CREB/BDNF pathway, protecting neurons and improving cognition. Created in BioRender. Jiejie, W. (2026) https://BioRender.com/necagsv.

Bidirectional communication between the gut and the brain depends not only on immune and inflammatory mediators but also on complex neural and molecular signaling pathways. The vagus nerve represents the most direct neural connection between the gut and the brain, and its afferent and efferent fibers play a central role in signal transmission within the MGBA (72). Increasing evidence indicates that the gut microbiota and its metabolites can actively influence brain structure, function, and plasticity through these neural pathways, as well as by modulating key host metabolic pathways (e.g., tryptophan metabolism), in a bottom-up manner (58). Disruption of these signaling mechanisms is critically involved in RBI(**Figure 2**).

### 3.1 Bidirectional regulatory role of the vagus nerve

The vagus nerve is the most direct neural pathway connecting the gut and the brain. Composed of afferent (sensory) and efferent (motor) fibers, it forms a complete perception–regulation circuit and serves as a core component of gut–brain axis signaling (41).

Vagal afferent fibers are responsible for sensing signals from the gut and transmitting them to the brain. These fibers can detect changes in microbial metabolites (e.g., SCFAs) and selectively activate vagal afferent neurons through receptor-mediated mechanisms (72). The transmitted signals are relayed via vagal afferent nuclei and ultimately influence higher-order brain functions. Experimental studies have demonstrated that electrical stimulation of vagal afferent fibers can directly modulate neurotransmitter levels in the brain, providing empirical support for the modulation of CNS states through this pathway (73).

Vagal efferent fibers mediate top-down regulation of the gut by the brain. Acetylcholine released from efferent terminals suppresses the release of pro-inflammatory cytokines, such as TNF-α, from M1-type macrophages in the intestinal lamina propria by activating the cholinergic anti-inflammatory pathway, thereby reducing local inflammatory responses (74, 75). This regulation of the intestinal immune environment can, in turn, influence the composition and function of the gut microbiota (76). Collectively, these mechanisms establish the vagus nerve as the functional basis for bidirectional gut–brain communication in RBI.

### 3.2 Central role of the tryptophan metabolic pathway

Beyond neural pathways, the gut microbiota also directly or indirectly influences signal homeostasis in the CNS by modulating key host metabolites. Among these, the metabolic pathway of the essential amino acid tryptophan is of particular importance. Tryptophan can cross the BBB, and its metabolism proceeds along two major branches that jointly regulate neuroactivity and immune balance. Certain gut microbes can metabolize tryptophan, thereby reducing its availability to the host (77).

Tryptophan serves as a precursor for the important neurotransmitter serotonin (5-hydroxytryptamine, 5-HT). In the brain, tryptophan is converted into 5-HT through hydroxylation and decarboxylation reactions. Serotonin is broadly involved in the regulation of mood, cognition, and neural repair (78). In parallel, the kynurenine pathway of tryptophan metabolism plays a central role in balancing neuroprotection and neurotoxicity. The intermediate metabolite kynurenine is taken up by different glial cell types in the brain, leading to divergent metabolic fates: astrocytes convert it into the neuroprotective metabolite kynurenic acid, whereas microglia may metabolize it into the neurotoxic compound quinolinic acid (78). Therefore, multilevel regulation of tryptophan metabolism by the gut microbiota represents a critical determinant of neuroimmune homeostasis in RBI.

### 3.3 Intervention and repair potential of signaling pathways

Positive modulation of the gut microbiota can activate protective signaling pathways within the CNS. Studies have shown that in irradiated mouse models, supplementation with total glucosides of paeony effectively modulates the gut microbiota, subsequently activating the cerebral cyclic adenosine monophosphate (cAMP)/cAMP response element-binding protein (CREB)/brain-derived neurotrophic factor (BDNF) signaling pathway. Activation of this pathway is associated with protection of hippocampal neurons and improvement in cognitive and spatial memory functions (79, 80). These findings demonstrate that interventions using prebiotics or microbiota-targeting drugs can activate beneficial neuroprotective and neuroplastic signaling pathways in a bottom-up manner.

Conversely, disruption of microbiota homeostasis leads to a series of adverse consequences. Evidence indicates that antibiotic-induced gut microbiota dysbiosis reduces the expression of genes associated with homeostatic microglial function (e.g., purinergic receptor genes P2ry12 and P2ry13) in the mouse spinal cord, while upregulating genes linked to neurodegenerative pathology (e.g., apolipoprotein E gene Apoe) (81). These findings suggest that an intact gut microbiota is essential for maintaining microglia in a homeostatic phenotype and supporting their normal physiological functions.

## 4 DNA damage repair dysfunction

Radiation exposure directly induces DNA double-strand breaks, leading to genomic instability and oxidative stress, and ultimately resulting in cell death (82). This DNA damage rapidly activates damage sensors such as ataxia-telangiectasia mutated (ATM) and ATM and Rad3-related (ATR), as well as downstream transcription factors including NF-κB, CREB, and activator protein-1 (AP-1). Activation of these pathways subsequently drives the expression of pro-inflammatory cytokines, such as interleukin-1β (IL-1β) and TNF-α. These systemic inflammatory responses may further disrupt intestinal barrier homeostasis and alter gut microbiota composition (4, 83).

Urolithin A (UroA), a gut microbiota–derived metabolite of ellagic acid, is associated not only with the restoration of intestinal microbial balance following radiation exposure but also with cellular protection through inhibition of p53-mediated apoptosis. UroA exerts protective effects on cellular DNA by suppressing NF-κB signaling, maintaining intracellular calcium homeostasis, and alleviating oxidative stress (84, 85).

## 5 Disruption of oxidative stress homeostasis

Ionizing radiation induces excessive production of reactive oxygen species (ROS) in the brain, resulting in neuronal and glial cell injury, mitochondrial dysfunction, and secondary DNA damage (86). Nuclear factor erythroid 2–related factor 2 (Nrf-2), a key endogenous transcriptional regulator of antioxidant responses, suppresses excessive ROS accumulation. Deficiency of Nrf-2 exacerbates radiation-induced injury and is accompanied by immune cell infiltration and elevated levels of pro-inflammatory cytokines (such as IL-6, IFN-γ, TNF-α), thereby disrupting gut microbiota homeostasis (87).

In parallel, the gut microbiota and its metabolites play an integral role in regulating oxidative stress within the brain. Experimental studies have demonstrated that exposure to 0.5 Gy radiation increases ROS levels in microglia, whereas exposure to 2 Gy induces hippocampal microglial activation and alters the activity of mitochondrial electron transport chain enzymes. These processes are regulated by the gut microbiota (55).

Protective microbial metabolites, including butyrate, as well as prostaglandin F2α and phenylacetylglutamine, can mitigate radiation-induced toxicity by reducing oxidative stress and enhancing mitochondrial function (88). In contrast, certain microbial metabolites, such as N^6^-carboxymethyllysine, may promote oxidative stress in microglia and impair mitochondrial function (89). Collectively, these findings indicate that gut microbiota–derived metabolites play a complex and pivotal role in RBI progression by modulating microglial redox balance and mitochondrial homeostasis.

## 6 Conclusions

Radiation therapy remains a cornerstone in the treatment of head and neck tumors; however, RBI in normal tissues continues to represent a significant unmet clinical challenge despite advances in treatment precision (90, 91). This review elucidates the role of the gut microbiota in brain injury, with a particular focus on bidirectional interactions mediated by the MGBA. We discuss how the gut microbiota modulates brain injury and repair through this axis and, conversely, how RBI reshapes gut microbial homeostasis. The underlying mechanisms primarily involve immune and inflammatory responses, signal transduction, DNA damage, and oxidative stress, all of which contribute to MGBA-mediated crosstalk. In addition, the potential application of deep learning models to elucidate the complex bidirectional regulatory roles of the MGBA in RBI is introduced.

Nevertheless, given the complexity of the biological mechanisms underlying RBI, a unified understanding of bidirectional MGBA communication—specifically, how injured brain tissue influences the gut microbiota and how the gut microbiota participates in brain repair—remains limited. Current studies on the interaction between RBI and the gut microbiota are relatively scarce and largely provide indirect evidence, underscoring the need for further investigation to elucidate precise mechanisms. As normal intestinal colonizers, gut microbiota are essential for host physiological homeostasis. RBI is frequently accompanied by gut microbiota dysbiosis, characterized by alterations in both bacterial abundance and diversity. Because distinct bacterial strains exert specific physiological functions, future studies should refine strain-level analyses and adopt interdisciplinary approaches integrating microbiology and immunology to support precision interventions for RBI. Furthermore, due to the presence of the BBB, communication between gut microbiota and brain tissue is typically indirect and primarily mediated by microbial metabolites or the vagus nerve. However, RBI is associated with BBB disruption, potentially enabling more direct microbial interactions with brain tissue. Current understanding of such interactions remains limited, highlighting the need for further systematic investigation. Alterations in the gut microbiota have the potential to feed back and modify brain function, as well as to influence recovery following injury. Such changes in the gut microbiota may serve as valuable biomarkers for monitoring disease progression, improving the prognosis of RBI, and even as therapeutic targets for addressing secondary injuries in patients with RBI. A comprehensive understanding of the complex direct and indirect interactions between the gut microbiota and RBI is therefore essential for the development of effective and targeted interventions to mitigate the deleterious effects of this condition.

Although the mechanisms underlying the gut microbiota–brain axis are complex and remain incompletely elucidated, the role of the gut microbiota and its metabolites should not be underestimated. The gut microbiota–derived metabolite indole-3-propionate (IPA) has been shown to promote sensory nerve axonal regeneration and functional recovery by recruiting neutrophils to the dorsal root ganglion. These findings indicate that microbial metabolites represent promising therapeutic candidates for nerve repair, axonal regeneration, and the enhancement of neurological recovery (92). However, whether the gut microbiota and its metabolites can serve as effective therapeutic targets for patients with RBI requires validation through well-controlled clinical trials in humans. In addition, the safety profiles of microbiota- and metabolite-based therapies warrant further systematic evaluation.

With ongoing advances in omics technologies and deep learning models, the translation of fundamental knowledge regarding the MGBA into therapeutic strategies for RBI is expected to become increasingly effective. The potential mechanisms discussed in this review, including radiation-induced effects on microglia, DNA damage, and ROS, may directly or indirectly influence the expression of inflammatory mediators, thereby affecting microbial homeostasis. Conversely, microbes and their metabolites can indirectly regulate immune factors, further modulating the progression of RBI. Given the complexity of these interactions, deep learning approaches are well suited to integrate multi-omics and multidimensional data, enabling interpretation from a more comprehensive and systematic perspective. Moreover, the rapid development of large language models, such as ChatGPT4, highlights the potential to integrate existing foundational knowledge of the MGBA to construct generalized brain–gut axis models, thereby facilitating the identification of potential interactions between gut microbes and brain tissue in RBI and revealing novel therapeutic avenues.

Collectively, a comprehensive understanding of the interplay between the gut microbiota and brain tissue in RBI may provide novel insights into radiation therapy, offer more effective strategies for future clinical practice, and ultimately improve the quality of life of patients affected by brain injury.
